# Evaluation of the Immunoprotective Potential of Recombinant Paraflagellar Rod Proteins of *Trypanosoma evansi* in Mice

**DOI:** 10.3390/vaccines8010084

**Published:** 2020-02-12

**Authors:** Biswa Ranjan Maharana, Naduvanahalli Rajanna Sudhakar, Vijayakumar Jawalagatti, Buddhi Chandrasekaran Saravanan, Damer P Blake, Anup Kumar Tewari

**Affiliations:** 1 Division of Parasitology, Indian Veterinary Research Institute, Izatnagar, Uttar Pradesh 243122, India; drbiswaranjanmaharana@gmail.com (B.R.M.); sudhi463@gmail.com (N.R.S.); vj734.vet@gmail.com (V.J.); drbcsaravanan@gmail.com (B.C.S.); 2 Department of Parasitology, Referral Veterinary Diagnostic & Extension Centre, Lala Lajpat Rai University of Veterinary and Animal Sciences, Hisar, Haryana 125001, India; 3 Animal Husbandry Unit, Government of National Capital Territory of Delhi, Delhi 110054, India; 4 Department of Pathobiology and Population Sciences, The Royal Veterinary College, Hertfordshire AL9 7TA, UK; dblake@rvc.ac.uk

**Keywords:** cytokine, immunization, mice, PFR, surra, *Trypanosoma evansi*

## Abstract

Trypanosomosis, caused by *Trypanosoma evansi*, is an economically significant disease of livestock. Systematic antigenic variation by the parasite has undermined prospects for the development of a protective vaccine that targets the immunodominant surface antigens, encouraging exploration of alternatives. The paraflagellar rod (PFR), constituent proteins of the flagellum, are prominent non-variable vaccine candidates for *T. evansi* owing to their strategic location. Two major PFR constituent proteins, PFR1 (1770bp) and PFR2 (1800bp), were expressed using *Escherichia coli*. Swiss albino mice were immunized with the purified recombinant TePFR1 (89KDa) and TePFR2 (88KDa) proteins, as well as with the mix of the combined proteins at equimolar concentrations, and subsequently challenged with virulent *T. evansi*. The PFR-specific humoral response was assessed by ELISA. Cytometric bead-based assay was used to measure the cytokine response and flow cytometry for quantification of the cytokines. The recombinant TePFR proteins induced specific humoral responses in mice, including IgG1 followed by IgG2a and IgG2b. A balanced cytokine response induced by rTePFR 1 and 2 protein vaccination associated with extended survival and improved control of parasitemia following lethal challenge. The observation confirms the immunoprophylactic potential of the covert antigens of *T. evansi*.

## 1. Introduction

Trypanosomosis or surra, caused by the unicellular hemoflagellate *Trypanosoma evansi,* is an economically significant disease of livestock and companion animals. The infection has wide prevalence in Asia, Africa, Central and South America, and parts of Europe [1,2,3,4,5]. The chronic form of the disease is common in South Asian cattle and buffalo, where it exerts a considerable economic impact. Infected livestock can suffer an increased risk of abortion, infertility, reduced milk yield, weight loss, and reduced draught capabilities [1,6,7]. Recent reports on *T. evansi* as a rare cause of zoonosis have raised concern for public health [8,9]. The disease is currently managed by chemotherapy with a small number of drugs available, viz. quinapyramine compounds, diminazene aceturate, suramin, and imidocarb dipropionate. Unfortunately, these drugs have undesirable side effects and leave metabolic residues in the liver and kidney, leading to necrosis of these organs [10]. Increasing evidence of emergence of drug resistant *Trypanosoma* strains has emphasized the need for a protective vaccine [11,12,13,14].

Surface proteins of *T. evansi* are highly immunogenic, but systematic antigenic variation by the parasite during infection has limited their value as vaccine candidates [15,16,17,18]. In response the invariant antigens, which are covert, have attracted attention as vaccine candidates against surra [10,19,20]. The paraflagellar rod (PFR) proteins are unique among the kinetoplastids and their heteropolymers form the building blocks of the flagellum [21]. The PFR proteins provide support for the metabolic regulators that may influence the flagellar movement of trypanosomes [22,23]. The structural dissimilarity of the kinetoplastid PFRs to proteins of mammalian cells, such as actin, tubulin, and intermediate filament proteins, reduces the risk of immunological cross-reactivity [24,25,26] and an autoimmune response [27]. The conserved nature of the kinetoplastid PFR genes offers the prospect of developing a vaccine against multiple *Trypanosoma* species. Here, we have used laboratory bred Swiss albino mice as hosts to test vaccine efficacy and responses. The mouse has been extensively used in experiments involving many *Trypanosoma* species, including *T. evansi* [1,7,15,20,28,29,30,31,32,33] and the model offers the convenience of checking post-inoculation parasitemia in vivo and the detection of antibodies in serum, studying the host factors that change the course of the disease and the genetic variation of the host that alters the severity of infection [34,35]. We report here use of recombinant paraflagellar rod proteins 1 and 2 to induce a partially protective immune response in experimental mice against *T. evansi*.

## 2. Materials and Methods

### 2.1. Parasite

A *Trypanosoma evansi* isolate, recovered previously from a horse and maintained as a cryostock, was used in the study [36].

### 2.2. Trypanosoma Evansi Whole Cell Lysate Antigen

The cryopreserved *T. evansi* was revived and propagated in vivo in Swiss albino mice by intra peritoneal inoculation as described elsewhere [37]. At the peak level of parasitemia, blood was collected from the heart under chloroform anesthesia. Trypanosomes were separated from the blood by DEAE-cellulose chromatography [37]. The purified parasites were pelleted by centrifugation at 4000× *g* at 4 °C for 5 min and washed with PBS (pH 7.2). The parasites were lysed by rapid freezing and thawing 5 times in liquid nitrogen, and were solubilized by ultra-sonication (Soniprep, Japan) at an amplitude of 15 for 30 s using 10 cycles on ice, with an inter-cycle interval of 30 s. Phenyl methyl sulfonyl fluoride (PMSF) was added to the cell suspension at a final concentration of 0.1 mM before sonication to avoid proteolytic denaturation. The trypanosome lysate was centrifuged at ~18,000× *g* at 4 °C for 30 min and the supernatant was retained. Following estimation of the protein concentration [38], the supernatant was aliquoted in 1ml volumes and stored at −20 °C until use.

### 2.3. Synthesis of *T. Evansi* cDNA

Total trypanosome RNA was extracted from purified *T. evansi* using Trizol reagent, following a standard protocol. Single-stranded complementary DNA (cDNA) was synthesized from total trypanosome RNA using an oligo dT primer protocol. Briefly, a 50 μL reverse transcription reaction was set up with 15 μL template RNA (4.5 μg), oligo dT primer 2 μL (100 pM), RT buffer (5×) 10μL, dNTPs (10 mM) 5 μL, RNase inhibitor (40 U/μL) 0.25 μL, MuMLV RT (200 U/μL) 2 μL, DEPC treated NFW 15.75 μL. The reaction was allowed to proceed for 1 h at 42 °C, following which the mixture was exposed to 70 °C for 10 min to inactivate the RT.

### 2.4. Prokaryotic Expression of TePFR1 and TePFR2

The full length TePFR1 open reading frame (ORF; 1770 bp) was PCR amplified using a specific primer pair containing restriction sites for *Nco*I and *Eco*RI (TePFR1NcoI-5’-ATC ACC ATG GCC GCA GTT GAC GAT GCCAC-3´ and TePFR1EcoRI-5´-GCT TGG AAT TCC TAT TCG AGG CGT GCC GGT GCA G-3´) with an annealing temperature of 57 °C. The specificity of the amplification was checked by agarose gel electrophoresis which resolved the TePFR1 specific 1770bp product. The PCR product was purified using a Qiagen MinElute Gel Extraction kit (Qiagen, Germany) following the manufacturer’s protocol. The PCR product was double digested in parallel with the pET-32a (+) expression vector using *Eco*R1 and *Nco*1 at 37 °C to facilitate directional cloning. The PFR 1 fragment was ligated to pET-32a (+) expression vector for transformation of competent *Escherichia coli* BL21 (DE3) cells by heat shock at 42 °C for 90 sec [36]. The cells were grown on LB agar containing ampicillin (100 µg/mL) at 37 °C overnight. The positive clones were grown overnight in LB broth containing ampicillin (100 µg/mL) at 37 °C with constant shaking at 140 rpm. The cells were induced with IPTG (1 mM). One ml of the uninduced culture was kept as control. The induced BL21 cell pellets collected at hourly intervals were analyzed by SDS PAGE (12%) to check the level of expression of the TePFR1 protein. Later, large cultures of 1 L volume were set up for production of bulk quantity of the recombinant protein.

Similarly, TePFR2 was expressed in *E. coli* following the same protocol. The full length ORF of TePFR2 (1800bp) was amplified from a *T. evansi* cDNA template by PCR using the specific primers Te PFR2 *Eco*RI-5´GGA ATT CAT GAG CGG AAA GGA AGT TGA AG 3´ and Te PFR2 *Hind*III-5´ CCC AAG CTT CTG AGT GAT CTG CGG CAT CGT G 3´.

### 2.5. Purification of Recombinant TePFR Proteins

The recombinant TePFR1 and TePFR2 proteins were purified by nickel affinity chromatography using a commercial purification kit as recommended by the manufacturer (Novagen, Inc., USA), followed by Triton X-114 phase separation to remove LPS contamination. The purity of the fusion proteins was checked by SDS-PAGE. The purified recombinant proteins were dialyzed against a decreasing concentration of urea at 6 h intervals and finally against PBS, pH 7.4 for 24 h at 4 °C for renaturation.

### 2.6. Western Blot Analysis of the Recombinant TePFR1 and TePFR2 Proteins

The recombinant TePFR1 and TePFR2 proteins were resolved by SDS PAGE and electrotransferred to nitrocellulose membrane. The identity of the histidine tagged recombinant TePFR1 and TePFR2 proteins were established by Western blot using anti-histidine monoclonal antibodies conjugated with HRP (Qiagen, Germany) following standard protocol [39].

### 2.7. Immunization Trial Using Recombinant TePFR1 and TePFR2 in Experimental Mice

#### 2.7.1. Experimental Design

The immunization trial was conducted in laboratory bred adult Swiss albino mice of either sex. The mice (*n* = 60) were randomly divided into six equal groups, Groups I to VI (Table 1).

#### 2.7.2. Parasitemia

Wet blood and Giemsa stained thin blood smears were examined microscopically daily to assess parasitemia post challenge. The trypanosomes were enumerated on a Neubauer’s counting chamber [40]. Data are presented as the arithmetic mean.

#### 2.7.3. Assessment of Humoral Immune Response

The humoral IgG, IgM, IgG_1_, IgG_2a_, and IgG_2b_ responses were assayed by ELISA post-immunization and challenge. Serum samples were collected at weekly intervals, and pooled group-wise to ensure sufficient starting volume for duplicate analysis by ELISA. The proteins used for immunization in each group were used as detection antigen to assay the humoral response for the respective groups. In brief, the rTePFR1 and rTePFR2 proteins were used as detection antigen for Groups I and II, respectively, whereas an equimolar mix of the rTePFR proteins was used to assess the humoral response in Group III. The TeWCL antigen was used to evaluate the humoral immune response in Group IV. The proteins were dissolved (5 µg/mL) in carbonate–bicarbonate buffer (pH 9.6) to coat the wells in 100 µL volume at 4 °C overnight. The wells were blocked with 200 µL fat-free milk powder (3% in PBS) (Ameresco, USA) for two hours at 37 °C. The test sera, diluted 1:100 in 1% non-fat milk powder in PBS, were loaded in duplicate wells and incubated for one hour at 37 °C. Subsequently, specific anti-mouse conjugates (Santa Cruz Biotech, USA), diluted 1:5000 in PBS containing 1% non-fat milk powder, were added and the plates were incubated for 1 h at 37 °C. The plates were washed stringently with PBS-T (Tween 20, 0.05%), pH 7.2 at every step of reaction. The wells were developed in darkness with 100 µL OPD substrate (0.4 mg/mL) (Sigma, USA) dissolved in citrate-phosphate buffer containing H_2_O_2_ (pH 5.0). Fifty microliter of 3N HCl was added to stop the reaction. The absorbance was recorded at 492 nm by an ELISA reader (Bio-Rad, USA).

To determine the end point titers of immunoglobulins on day 35 of the experiment (Day7 PC), the pooled sera from different treatment groups were serially diluted two-fold, starting from 1:50 to 1:800. Sera samples (*n* = 60) collected from the pre-immunized mice on day 0 formed six control pools. The cut-off value was determined by adding 3 standard deviations (3SD) to mean OD_492_ values of healthy mice sera diluted 1:100. The titer of the immunoglobulins represented the specific dilution of sera at which the OD_492nm_ value was higher than the cut-off value. 

#### 2.7.4. Assessment of Cytokine Response

Multiple cytokines, including TNF-α, IFN-γ, IL-10, IL-4, IL-5, and IL-2, were simultaneously detected in the mouse serum by bid array based BD CBA Mouse Th1/Th2 Cytokine kit following the manufacturer’s protocol (BD Biosciences, USA). The group-wise pooled sera samples, collected at weekly intervals, were used for the assay. The mixed bead standard, provided in the kit, was used to generate the standard curve for each cytokine.

### 2.8. Statistical Analysis

The data were derived from at least triplicate observations for each sample per time-point and expressed as Mean ± SEM. The data were analyzed statistically by analysis of variance (ANOVA) using Duncan’s multiple range test using SPSS 11.0 software and the *p*-value ≤ 0.05 denoted significant difference.

### 2.9. Ethics Statement

The experiments involving laboratory animals were carried out conforming to the ethical considerations and guidelines of the Indian Veterinary Research Institute and with prior approval of the Institute Biosafety Committee (IBSC) and Institute Animal Ethics Committee (IAEC) (Reference F-26-1/201516/JD(R)).

## 3. Results

### 3.1. Heterologous Expression and Purification of rTePFR1 and rTePFR2

The complete coding sequences (CDS) of TePFR1 and TePFR2 were PCR amplified from *T. evansi* cDNA (Supplementary Material, Figure S1a) using specific pairs of primers and were sequenced for confirmation (GenBank: FJ968743.1; GenBank: FJ901341.1). High level expression of rTePFR1 and rTePFR2 proteins was recorded after 4 and 6 h of induction of the *E. coli* cells with IPTG, respectively. The proteins were resolved at the Mr of 89 and 88 kDa, respectively by SDS-PAGE (Supplementary Material, Figure S1b). The histidine tagged recombinant TePFR1 and TePFR2 proteins were identified by their immunoreactivity to anti-histidine monoclonal antibodies conjugated with HRP (Qiagen, Germany) by western blot at 89 and 88 kDa region, respectively (Supplementary Material, Figure S1c,d).

### 3.2. Survival/Mortality of Mice Post Challenge

The time taken for establishment of the parasites in the immunized mice and the extent of their survival were used to determine the extent of protection post-challenge (PC).

**Figure 1:**
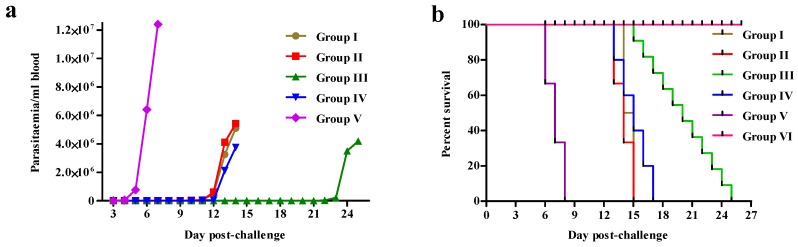
(**a**) The time course of parasitemia in different groups of mice post challenge. (**b**) Mouse survival following challenge by 1 × 10^2^ *T. evansi* blood stream forms. Mice were immunized with rTePFR1 (Group I), rTePFR2 (Group II), an equimolar mixture containing rTePFR1 and rTePFR2 (Group III), *T. evansi* whole cell lysate (TeWCL) (Group IV) or FCA alone (Group V). Group VI was unimmunized and unchallenged. The boosters were administered on days 7 and 21. The mice were challenged with 1 × 10^2^ live *T. evansi* through the intraperitoneal route on day 28 (shown here as 0 days post challenge). Parasitemia was monitored from day one post-challenge till death of the mice by counting trypomastigote forms in the peripheral blood obtained by sampling from the tail. Mean values (*n* = 10) ± SEM are shown.

Parasites were first detected in the peripheral blood of the non-immunized mice of the challenge control group (Group V) on day 3 PC and mortality of the mice in this group was complete by day 8 PC (Figure 1a,b). The onset of parasitemia in the immunized mice of Groups I and II was delayed and occurred on day 9 PC. Two mice from Group I were blood smear positive on day 9 PC. The initial low parasitemia (1.25 × 10^4^/mL) eventually increased to 3.25 × 10^6^/mL by day 13 PC. The remaining eight mice from this group showed comparable parasitemia on day 14 PC. Fifty percent of the mice from Group I and forty percent from Group II survived up to day 14 PC, but the remaining mice succumbed to infection by day 15 PC. Parasites were detected in the blood first on day 19 PC (7.5 × 10^3^/mL) in Group III (recombinant PFR1 and PFR2 mix). Thirty percent of the mice from this group survived until day 21 PC, while the remaining mice survived up to day 25 PC. Parasitemia was first recorded in the TeWCL immunized mice (Group IV) on day 9 PC (8.75 × 10^3^/mL) and all of the mice from this group died by day 17 PC.

### 3.3. Humoral Response Following Immunization and Challenge

**Figure 2:**
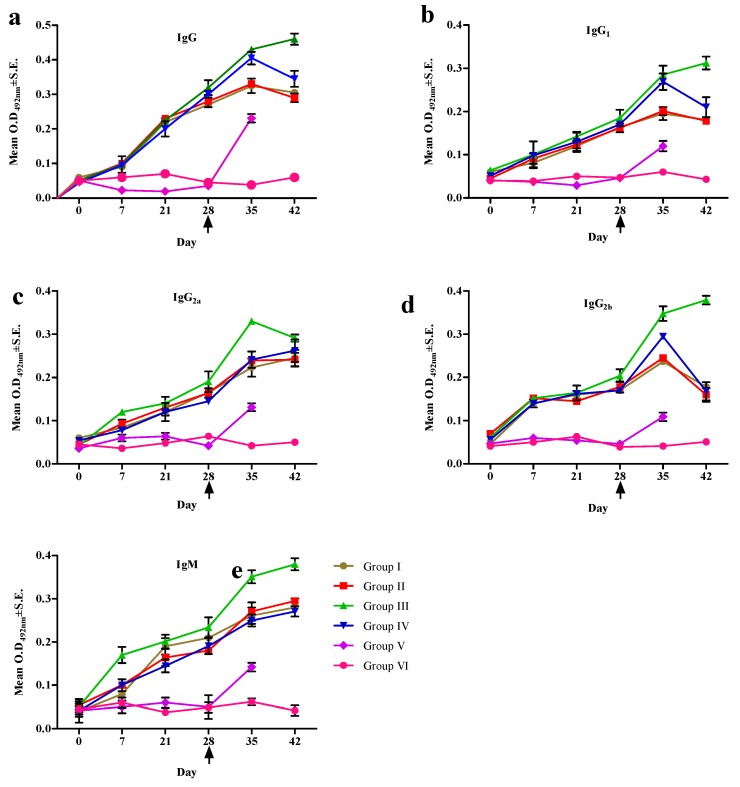
The serum profile of IgG (**a**), IgG_1_ (**b**), IgG_2a_(**c**), IgG_2b_ (**d**), and IgM (**e**) in mice following immunization with recombinant TePFR proteins and challenge with live *T. evansi* (arrow). rTePFR1, rTePFR2 and an equimolar mixture of rTePFR1 and rTePFR2 were used as detection antigen for the indirect ELISA to assess the specific antibody responses in the respective treatment groups (Group I, Group II, and Group III, respectively). We compared the humoral response induced by rTePFR with that of the native *T. evansi* whole-cell lysate antigen (TeWCL) immunized group and used the native TeWCL antigen in ELISA for Group IV. Data presented as Mean ± SEM from the surviving mice in each group. Note, day 42 (equivalent to 14 days post-challenge) is the last data point presented, after which mortality begun to undermine representative group sizes. The arrow denotes the day of challenge.

**Figure 3:**
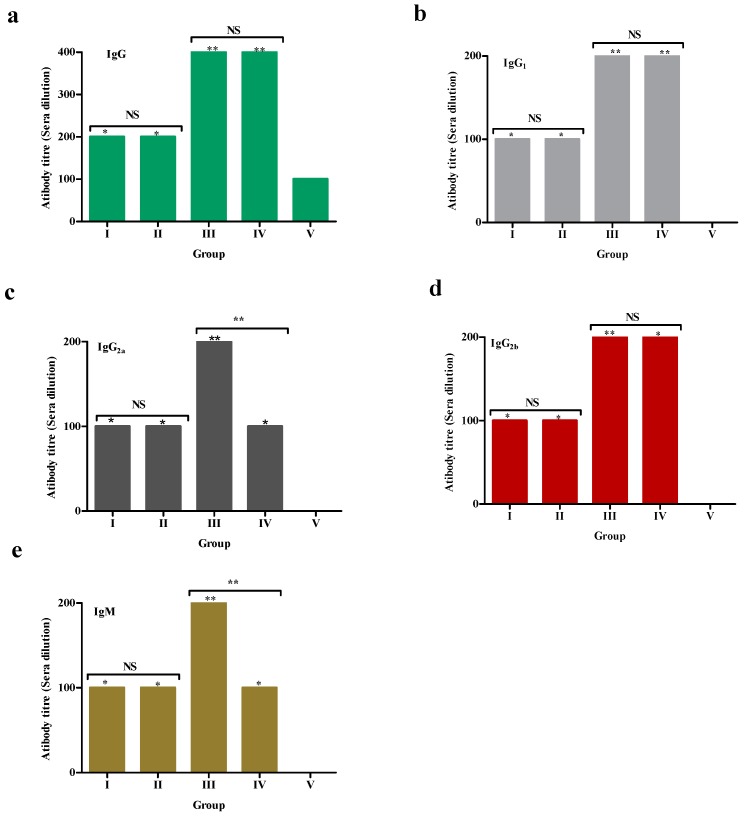
Serum antibody titer of IgG (**a**), IgG_1_ (**b**), IgG_2a_(**c**), IgG_2b_ (**d**), and IgM (**e**) recorded in different groups of experimental mice following virulent *T. evansi* challenge. The serum antibody titer was determined in different treatment groups on day 7 post-challenge. The pooled sera samples were serially double diluted and tested by indirect ELISA. The values for different treatment groups were compared. The ELISA was performed using the group-specific proteins used for immunization, as detection antigen. The asterisks indicate a significant differences between groups (* *p* < 0.05, ** *p* < 0.001, ^NS^*p* > 0.05). The cut-off values were calculated by adding 3SD to mean OD_492_ of healthy control mice and were 0.205, 0.185, 0.172, 0.216, and 0.235, respectively, for IgG, IgG_1_, IgG_2a_, IgG_2b_, and IgM.

The recombinant TePFR proteins induced specific humoral responses post-immunization in the experimental mice (Figure 2 and Figure 3). The peak IgG response was recorded on day 35 in mice of Groups I, II, and IV as 0.325 ± 0.021 (1:200 titer), 0.33 ± 0.004 (1:200 titer), 0.405 ± 0.018 (1:400 titer) respectively, whereas, in Group III the peak response was recorded on day 42 (0.46 ± 0.016, 1:400 titer). Similarly, the peak IgG_1_ response was detected on day 35 in Group I (0.195 ± 0.015, 1:100 titer), Group II (0.201 ± 0.009, 1:100 titer) and Group IV (0.269 ± 0.019, 1:200 titer). The peak IgG_1_ response was recorded on day 42 (0.312 ± 0.015, 1:200 titer) in Group III, whereas, the IgG_2a_ response was highest at day 35 (0.33 ± 0.007, 1:200 titer). A significantly higher IgG_2b_ response was recorded in the mix antigen vaccinated group (Group III) on day 14 PC (0.379 ± 0.023, 1:200 titer) of the experiment compared to the other immunized groups (*p* ≤ 0.05).

An IgM response was detected by day 7 PI and the peak response was recorded in all immunized groups on day 7 PC, declining after that until day 14 PC. In Group III (mix of proteins immunized), the IgM concentration increased with the peak observed on day 14 PC (0.38 ± 0.032, 1:200 titer) of the experiment (*p* ≤ 0.05) (Figure 2 and Figure 3).

### 3.4. Serum Cytokine Responses Following Immunization and Challenge

A gradual increase in the serum concentration of TNF-α and IFN-γ was recorded in the immunized mice from day 7 post immunization (PI) onwards, and more rapidly in the unimmunized control group post challenge (PC). The peak serum concentration of TNF-α was recorded on day 42 as 126.6 ± 1.2, 102.45 ± 1, and 117.35 ± 2.35 pg in Groups I, II, and IV, respectively, whereas the peak was recorded on day 35 in the challenge control group (Group V; 102.5 ± 0.7 pg/mL). The peak serum TNF-α concentration was recorded on day 35 in Group III (rTePFR1 + rTePFR2 proteins immunized; 92.85 ± 0.45 pg, which declined by day 42 (*P* ≤ 0.05).

The serum IFN-γ concentration increased post challenge and the maximum level was recorded on day 42 at 136.425 ± 1.42, 134.6 ± 0.6 and 136.25 ± 10 pg/mL, in Groups I, II, and IV respectively. The peak serum IFN-γ concentration was recorded on day 35 in Group III (71.22 ± 0.22 pg, and the level declined after that (*p* ≤ 0.05). In Group V, the peak serum IFN-γ concentration was recorded on day 35 (143.09 ± 2.42 pg/mL).

**Figure 4:**
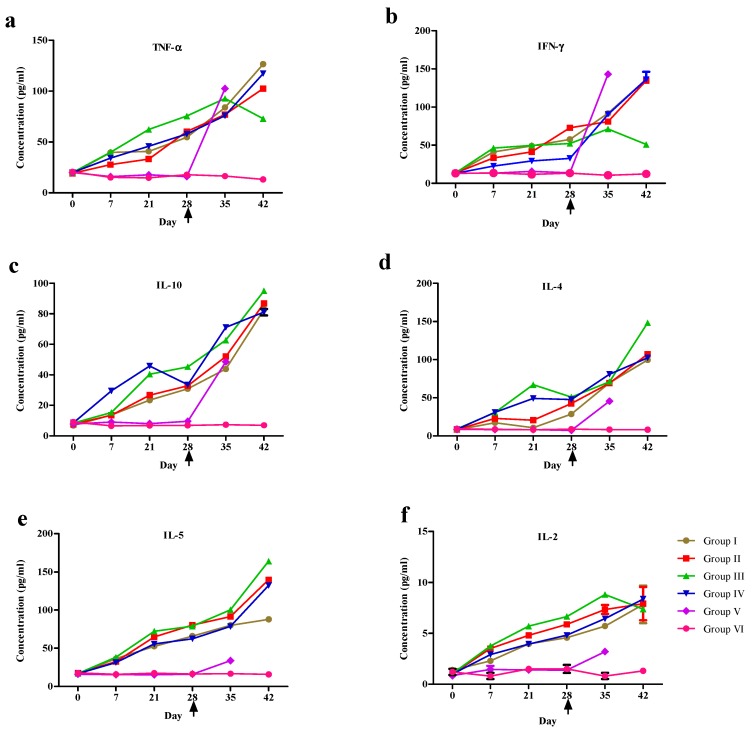
Kinetics of serum TNF-α (**a**), IFN-γ (**b**), IL-10 (**c**), IL-4 (**d**), IL-5 (**e**), and IL-2 (**f**) in rTePFR (Groups I to III), or the adjuvant administered control mice (Group V) on days 0, 7, and 21. The mice were challenged with *T. evansi* blood-stream forms on day 28 (arrow). The response was compared with that of the native *T. evansi* whole cell lysate antigen immunized group (Group IV). Data presented as Mean ± SEM from the surviving mice in each group. Note, day 42 (equivalent to 14 days post-challenge) is the last data point presented, after which mortality begun to undermine representative group sizes.

A moderate but statistically significant (*P* ≤ 0.05) increase in the serum concentration of IL-10 was recorded in Group III. A progressive post immunization increase in the serum concentration of IL-4 and IL-5 was recorded and the peak serum concentration was recorded on day 42 as 148.26 ± 2.06 pg/mL and 164.1 ± 1.1 pg/mL, respectively. The increase was statistically significant in Group III for IL-4 and IL-5 (Figure 4a–f). A modest increase in the concentration of IL-2 was observed in all immunized groups except in the rTePFR-mix group, where a declining trend was observed on day 42 (*p* ≤ 0.05).

## 4. Discussion

Immune evasion by systematic antigenic variation, a hallmark of trypanosoma infection, has thwarted efforts to develop protective vaccines targeting immunodominant surface glycoproteins. The molecular basis of antigenic variation in *Trypanosoma evansi* is known to a large extent, but a foolproof strategy for blocking or responding to the sequential expression of variable antigenic surface epitopes in vivo has not yet been successful. Therefore, identification of non-variable proteins as vaccine targets has been a priority. Structurally important hidden antigens such as the paraflagellar rod proteins, beta tubulins and flagellar pocket antigens, are promising vaccine targets [15,20,28,41,42]. The free flagellum contributes to trypanosome motility and is essential for their survival in the host blood and tissue fluid. Once established in its host the parasite proliferates rapidly, leading to pathological consequences [43,44]. Heteropolymers of the kinetoplastid paraflagellar rod (PFR) proteins [45] are the building blocks of the flagellum [21]. Paraflagellar rod 1 and 2 (PFR1 and PFR2) proteins are important components of the flagellum ultrastructure. Unlike the broadly conserved eukaryotic axoneme, the PFR is restricted to kinetoplastids, euglenoids, and dinoflagellates [46,47,48] and has emerged as a promising drug target [49]. Earlier, the genes coding for *T. brucei* paraflagellar rod proteins PFRA and PFRC were identified and the proteins were purified [50,51]. The orthologues of these molecules were reported from related kinetoplastids *Trypanosoma* and *Leishmania* only [25,52,53,54].

We achieved a high level of expression of the recombinant *T. evansi* PFR proteins 1 and 2 in *E. coli*. The proteins were affinity purified and their identity was confirmed by western blot using anti-histidine-HRPase conjugate. Further, we noted an absence of cross-reactivity between the rTePFR1 and 2 proteins by western blot using specific hyper immune sera raised in New Zealand white rabbits. The immune response induced by the rTePFR proteins in Swiss albino mice was compared to that of native *T. evansi* whole cell lysate (TeWCL). Serum was isolated after tail bleeding individual mice, however the minute quantity isolated from each individual necessitated samples be pooled to provide sufficient material for detailed analysis.

The rTePFR1 and rTePFR2 proteins induced humoral IgG responses comparable to that of the whole cell lysate. Combined, the rTePFR proteins induced an earlier IgG response that peaked on day 42. The rTePFR1 and 2 proteins induced an IgG_1_ response when used alone and the peak response was noted on day 35. Immunization using the mixed rPFR proteins was associated with a peak response seven days later. A role of IgG_1_ and IgG_2_ antibodies in protection against trypanosomes has been reported by others [55]. Previous vaccination trials in mice showed that IgG_1_ was associated with a Th2-like response, while a Th1 response was associated with the induction of IgG_2a_, IgG_2b_, and IgG_3_ antibodies [56]. IgG_2a_ and IgG_2b_, together with IgG_3_, bind strongly to Fc receptors and can fix complement better than IgG_1_. While circulating antibodies commonly mediate effector immune mechanisms against extracellular pathogens like trypanosomes, a combination of both T and B-cells are required for an effective response [19]. Although we did not make any attempt to quantify the concentration of the PFR1 and 2 proteins in the native *T. evansi* whole cell lysate preparation, the rTePFR immunized mice showed immunoreactivity to the native protein in ELISA (data not presented). The processing of immunolyzed parasites might be the source of otherwise hidden PFR proteins inducing a polyclonal activation.

Quantifying circulating levels of cytokines can provide important information on the pathogenesis of disease [57,58]. The broad dynamic range of fluorescence detection via flow cytometry and the efficient capturing of analytes via suspended particles enable the BD CBA assay employed here to measure the concentration of an unknown in substantially less time and using fewer sample dilutions than a conventional ELISA methodology. TNF-α has previously been associated with control of trypanosome parasitemia, reducing infection associated pathology and mediating resistance during infection [59]. The soluble variable surface glycoproteins (VSGs), shed by live trypanosomes are thought to be the major TNF-α inducers [60,61], and an increasing TNF-α concentration has been linked to a failing immune system and uncontrolled infection [62]. Similar observations have been recorded elsewhere [7,28,42,59,63,64]. However, the elevated serum concentration of TNF-α in the TePRF1/2 co-immunized mice possibly indicates a role in better protection and extended survival [64].

Serum IFN-γ was detected at a significantly higher concentration in non-immunized mice post-challenge when compared to their immunized counterparts (*p* < 0.05). T- and natural killer (NK) cells primarily secrete IFN-γ, which plays a vital role in innate immunity due to its pro-inflammatory properties and acts as an inducer of the adaptive immune response. The IFN-γ induced macrophages generate trypanocidal molecules including reactive oxygen intermediates, reactive nitrogen intermediates and TNF [65]. The crucial role for IFN-γ in controlling *T. b. brucei* and *T. b. rhodesiense* parasitemia has been documented in mice [66,67,68]. Infection of IFN-γ knockout mice results in earlier, uncontrolled parasitemia leading to significantly reduced survival [67]. In contrast, epidermal growth factors mediate stimulation of parasite growth by IFN-γ was reported by Olsson et al. (1991) [69]. A gradual decrease in the serum concentration of IFN-γ and IL-2 might have beneficial effect on the host.

The increased serum concentration of IL-10, IL-4, and IL-5, prominent cytokines in the Th2 pathway, was indicative of a balanced T-helper cell response in the immunized mice. IL-4 has a role diverting T-helper cells towards a Th2 response, while IFN-γ inhibits IL-4 and its effects. Early regulation of cytokines in the Th1 and Th2 pathways helps avoid a polarized immune response with a cascade effect. The IL-10, IL-4, and IL-5 mediated Th2 response might have been crucial in containing the surge of TNF-α and IFN-γ in the immunized groups. This balancing act of the Th2 cytokines to partially ameliorate the effects of the Th1 response on the host might have helped control progression towards a fatal outcome, significantly prolonging the life span of the immunized mice [61,70,71,72].

A detectable IgM response was recorded throughout the observation period, albeit at a low titer, and might have played a crucial role in the humoral protective response. The onset of parasitemia in the peripheral blood was delayed in mice immunized with the mixture of the rTePFR proteins. The rTePFR protein mix had the advantage of plurality of determinants of the PFR antigen over immunization with the individual rTePFR proteins.

## 5. Conclusions

Immunizing Swiss albino mice with rTePFR proteins induced a balanced Th-cell response as evidenced by circulating blood Th1 and Th2 cytokine profiles, as well as a humoral IgG isotype response. Although the immune response elicited by the rTePFR proteins could not save the mice following virulent *T. evansi* challenge, the survival period of the immunized mice extended significantly post-challenge. The protection may be attributed to the higher serum concentration of IgG_2a_ and a balance of the pro-inflammatory cytokines TNF-α and IFN-γ. These observations reassert the potential of covert PFR antigens as viable vaccine targets capable of generating protective cell mediated and humoral immune responses against the lethal hemoparasite challenge. Further studies on the establishment of an optimal balance between Th1 and Th2 responses through selection of the appropriate route of delivery, an adjuvant and immunization schedule might help in inducing a solid protection.

## Figures and Tables

**Table 1:** Experimental design to assess the immunoprotective potential of rTePFR proteins in mice. The mice in Groups I to III were immunized with 30 μg of recombinant rTePFR, either singly or in combination, through intramuscular injection in the thigh muscle. The mice in Group IV were immunized with *Trypanosoma evansi* whole cell lysate (TeWCL) antigen. The proteins were emulsified in FCA (total volume 100 μL) for primary immunization. The boosters comprised of the proteins emulsified in Freund’s incomplete adjuvant in equal ratio. The mice in Group V were inoculated with the adjuvant alone and served as unimmunized infection controls. The mice were challenged with virulent *T. evansi.* Mice in Group VI served as healthy controls (unimmunized, unchallenged).

Group. (10 mice per group)	Treatment	Schedule and Route of Inoculation	Challenge Inoculum and Route of Inoculation
I	rTePFR1	30 µg on day 0, 7, and 21 Intramuscular, thigh muscle	1 × 10^2^ on day 28 Intraperitoneal
II	rTePFR2		
III	Equimolar mixture of rTePFR1 and 2		
IV	TeWCL		
V	Adjuvant control	Adjuvant only	
VI	Healthy control	----	----

## Supplementary Materials

The following are available online at https://www.mdpi.com/2076-393X/8/1/84/s1, Figure S1a: Specific primer directed PCR amplification of the full ORF of *T. evansi* TePFR1 and TePFR2. Lane M: 100 bp plus DNA marker, Lane 1: Amplified 1800bp full length nucleotide sequence of TePFR2, Lane2: Amplified 1770 bp full length nucleotide sequence of TePFR1; Figure S1b: The rTePFR1 and rTePFR2 proteins were purified by Ni-NTA affinity chromatography. 12% polyacrylamide gel showing the resolution of rTePFR1 and rTePFR2 at 89kDa and 88kDa region, respectively; Lane M: Pre-stained protein molecular weight marker (Invitrogen), Lane 1: Purified TePFR1 protein, Lane 2: Purified TePFR2 protein; Figure S1c: Western blot showing reactivity at 89 kDa for rTePFR1 protein at Lane 1. Lane M: Pre-stained protein molecular weight marker; Figure S1d: Western blot showing reactivity at 88kDa for rTePFR2 protein at Lane 1. Lane M: Pre-stained protein molecular weight marker.
