# Supplementary material for: Evaluation of the Immunoprotective Potential of Recombinant Paraflagellar Rod Proteins of Trypanosoma evansi in Mice
Source: Vaccines (Basel). 2020 Feb 12;8(1):84. doi: 10.3390/vaccines8010084 (PMC7157580; doi:10.3390/vaccines8010084)
Supplement: Supplementary file 1 [file vaccines-08-00084-s001.pdf]

vaccines

Supplementary Material

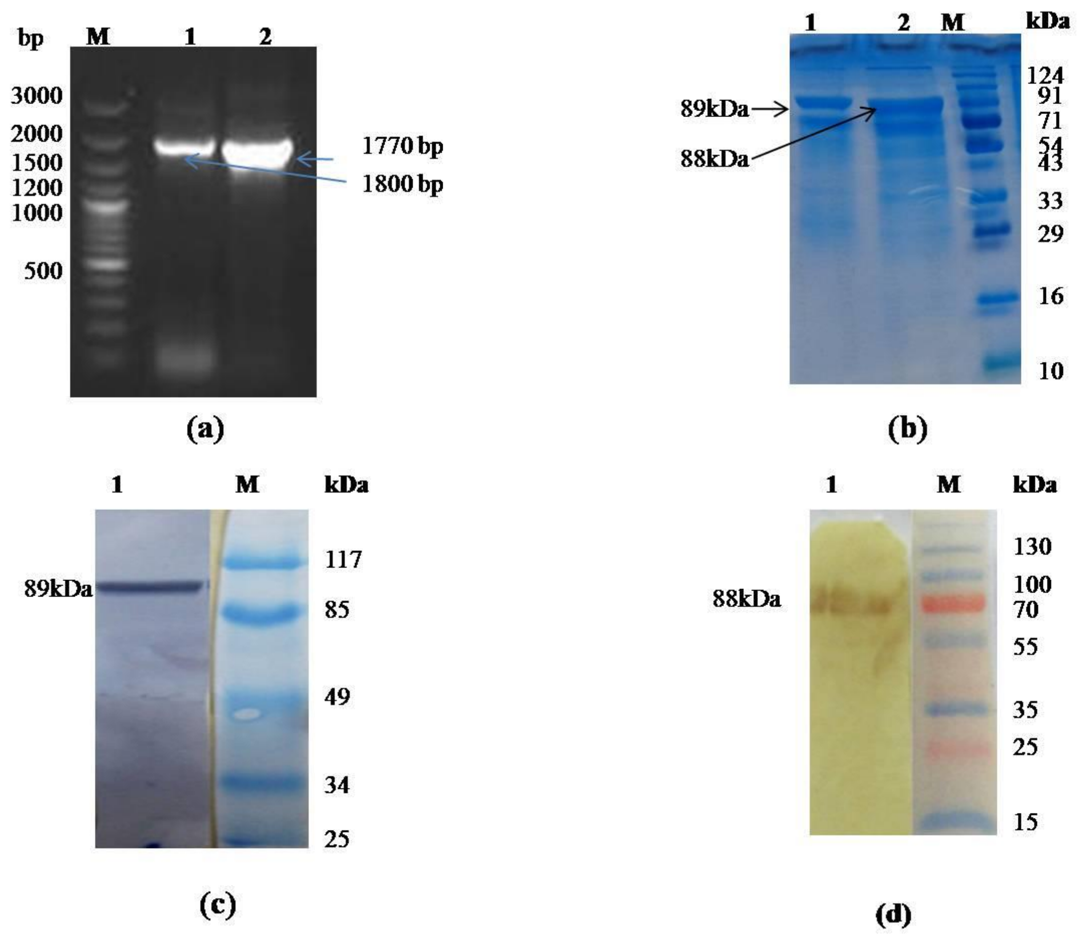

Supplementary Figure S1

(a) Specific primer directed PCR amplification of the full ORF of *T.*evansiTePFR1 and TePFR2. Lane M: 100 bp plus DNA marker, Lane 1: Amplified 1800bp full length nucleotide sequence of TePFR2, Lane2: Amplified 1770 bp full length nucleotide sequence of TePFR1;

(b) The rTePFR1 and rTePFR2 proteins were purified by Ni-NTA affinity chromatography. 12% polyacrylamide gel showing the resolution of rTePFR1 and rTePFR2 at 89kDa and 88kDa region, respectively; Lane M: Pre-stained protein molecular weight marker (Invitrogen), Lane 1: Purified TePFR1 protein, Lane 2: Purified TePFR2 protein;

(c) Western blot showing reactivity at 89kDa for rTePFR1 protein at Lane 1. Lane M: Pre-stained protein molecular weight marker.

(d) Western blot showing reactivity at 88kDa for rTePFR2 protein at Lane 1. Lane M: Pre-stained protein molecular weight marker.
